# Evaluation of Methods to Obtain Peripheral Blood Mononuclear Cells From Deceased Donors for Tolerance-Induction Protocols

**DOI:** 10.1177/09636897241256462

**Published:** 2024-05-29

**Authors:** Ming Yao, Jarmo Henriksson, Henrik Fahlander, Pablo Guisti Coitinho, Torbjörn Lundgren, Nils Ågren, Bo-Göran Ericzon, Makiko Kumagai-Braesch

**Affiliations:** 1 Division of Transplantation Surgery, CLINTEC, Karolinska Institutet, and Department of Transplantation Surgery, Karolinska University Hospital, Huddinge, Stockholm, Sweden; 2 Centre for Apheresis and Stem Cell Laboratory, KITM, Karolinska University Laboratory, Karolinska University Hospital, Stockholm, Sweden

**Keywords:** T-cells, surgery, immunomodulation, cellular therapy

## Abstract

Regulatory cell therapies have shown promise in tolerance-induction protocols in living donor organ transplantation. These protocols should be pursued in deceased donor transplantation. Donor peripheral mononuclear cells (PBMCs) are an optimal source of donor antigens for the induction of donor-specific regulatory cells. During the development of a regulatory cell tolerance-induction protocol with organs from deceased donors, we compared 3 methods of obtaining PBMCs from deceased donors focusing on cell yield, viability, and contamination of unwanted cell types.

PBMC procurement methods:

1. During organ procurement at the time of cold perfusion, blood was collected from the vena cava and placed into a 10-liter blood collection bag, and thereafter transported to Karolinska University Hospital, where leukapheresis was performed (BCL).

2. Blood was collected via the vena cava into blood donation bags before cold perfusion. The bags underwent buffy coat separation and thereafter automated leukocyte isolation system (BCS).

3. To collect PBMCs, leukapheresis was performed via a central dialysis catheter on deceased donors in the intensive care unit (ICU) prior to the organ procurement procedure (LEU).

All 3 methods to obtain PBMC from deceased donors were safe and did not affect the procurement of organs. BCL contained around 50% of NK cells in lymphocytes population. LEU had a highest yield of donor PBMC among 3 groups. LEU had the lower amount of granulocyte contamination, compared to BCS and BCL. Based on these results, we choose LEU as the preferred method to obtain donor PBMC in the development of our tolerance-induction protocol.

## Introduction

Long-term allogenic graft acceptance without chronic immunosuppression (IS) has been desired since the birth of clinical transplantation. In the last 2 decades, experimental protocols that induce tolerance have successfully led to the minimization of IS and, in the most successful cases, complete abolishment of the need for lifelong IS. This has moved the concept of tolerance induction closer to clinical reality^1–5^. There are several strategies to achieve tolerance after allogenic transplantation.

Inducing a state of transient or permanent donor-specific chimerism in the recipient with a hematopoietic stem cell transplant has shown promise in living-donor kidney transplantation^2,3,6^ but often requires at least a partial myeloablative induction regimen^7^, a procedure which is invasive and may not be suitable for all transplant recipients.

Regulatory cell therapies have emerged as a less-invasive strategy and have shown encouraging results with complete withdrawal of IS in living-donor liver transplantation^5,8^, and minimizing IS in living-donor kidney transplantation^4,9^. Although the regulatory T-cell (Treg) is the presumed protagonist in the immunomodulatory mechanisms that lead to tolerance induction, other suggested regulatory cell lines are emerging^10–12^. The starting material in the manufacturing process of such cell therapy products is most often derived from the recipient. Alloantigen-specific regulatory cells (induced regulatory cells) are more effective than polyclonal or naturally occurring regulatory cells of the recipients^13,14^. Hence, donor antigens are required for the stimulation of the recipient start material required if a donor-specific cell product is to be generated. Certain steps in this production process, such as antigen stimulation, are considered manipulation and classifies the cell product as an advanced therapeutics medicinal product (ATMP), where strict cGMP guidelines regarding sterility and mitigation of transmission of pathogens must be adhered to throughout the manufacturing process^15^, including the procurement of recipient and donor cells.

In the elective living-donor transplantation setting, the procurement of donor starting material can be scheduled ahead of the donation and transplantation procedure. However, in most Western countries, most solid organs used for transplantation are obtained from deceased donors^16^. In such cases, the planning and execution of the transplantation procedure often occurs with a short timeline and outside office hours. Unlike procuring recipient-derived starting material that can be performed immediately before the transplantation procedure, obtaining the necessary quantity of donor-specific antigens can be technically and logistically challenging because many organ procurement procedures are performed outside office hours in distant hospitals.

With recent advancements in using regulatory cells for tolerance-induction therapies in living-donor transplantation^4,5,8^, evaluating and enabling these treatment options for recipients of deceased donor transplants is a crucial next step. Our center is developing a tolerance-induction protocol using donor-specific immunomodulatory cell therapy for recipients of deceased donor liver transplantation. As a part of the preparatory work, in this study, we evaluate 3 methods of obtaining peripheral mononuclear cells (PBMCs) from deceased donors, focusing on total cell yield, viability, and cell composition in a nonelective situation.

1. Blood procurement during cold perfusion of the organ donor followed by leukapheresis of the procured blood at the transplant center—BCL.
2. Blood collection before cold perfusion and PBMC isolation using automated leukocyte separation system (ALSS) at the transplant center—BCS.
3. Bedside leukapheresis of the donor prior to organ procurement—LEU.

## Material and Methods

### Ethical Considerations

The procurement of blood from deceased donors and leukapheresis in the intensive care ward prior to the organ procurement procedure were reviewed and approved by the Swedish Ethical Review Authority (EPN) nr: 2014/1565-31/4 and 2019-04445.

### Deceased Donors

Deceased organ donors within the region of Stockholm and the Uppsala district approved for “donation for transplantation or other medical purposes” were enrolled in this study. All included donors were donation after brain death (DBD) donors. Peripheral blood samples were taken after brain death and analyzed for hemoglobin, white blood cells (WBCs), and C-reactive protein (CRP).

#### Blood procurement during cold perfusion of the organ donor followed by leukapheresis of the procured blood at the transplant center—BCL

During organ procurement, after the cannulation of the aorta or iliac artery for cold perfusion, the 30Fr tubing of a 10-liter blood collection bag (Thermo-Fisher, Waltham, Massachusetts, USA) was secured in the abdominal vena cava and fixated with Vicryl (Ethicon, Raritan, New Jersaey, USA) ligatures. Heparin 400 EQ/kg was given systemically according to standard procedure. During the cold perfusion, the blood, including the perfusion solution Institut Georges Lopez-1 (Institut Georges Lopez, Lisseu, Rhone-Alpes, France) was drained into the blood collection bag containing 750 mL anticoagulant citrate-dextrose solution (ACD) (Terumo BCT, Colorado, USA). The aim was to obtain as high a blood volume as possible. The blood collection bag was sealed after the end of blood procurement and transported to Karolinska University Hospital at 2°C to 6°C.

The bag was connected to a Spectra Optia, (Terumo BCT, Colorado, USA) system, and leukapheresis was performed by using local protocol (continuous mononuclear cell collection [CMNC] protocol, following standard operating procedure (SOP): IMT3127 Karolinska University Hospital). The leukapheresis product was analyzed for cell count, viability, and composition using flow cytometry (FACS) (see method below).

#### Blood collection before cold perfusion and PBMC isolation using automated leukocyte isolation system at the transplant center—BCS

During the organ procurement procedure, just before the heparinization and cannulation of the aorta for cold perfusion, the abdominal vena cava was encircled with Vicryl ligatures. One standard blood donation bag NPT6280LE Quintruble Bag LCRD-S (Maco Pharma, Tourcoing, France) was connected via syringe to the vena cava and blood was drained into the bag through gravity. Rocking of the bag was performed during the filling to mix the blood with citrate-phosphate-dextrose (CPD) (Sigma-Aldrich St. Louis, Missouri, USA). The bag was filled to 775 to 875 gm and clamped, a second bag was subsequently connected to the vena cava and the process was repeated until blood flow diminished, or blood pressure decreased.

The blood bags were transported to Karolinska University, and the buffy coat was separated out by using a Macospin (Maco Pharma, Tourcoing, France) centrifuge following SOP IMT0420 Karolinska University Hospital. Leukocytes were further purified using ALSS (SEPAX, Biosafe, East Hartford, Connecticut, USA) in a closed sterile system (ref. CS-900.2) with a gradient centrifugation protocol (Neatcell v314). Starting volumes of up to 120 ml were processed using a gradient solution (Ficoll-Paque™ PREMIUM Ref 17-5442-02, GE Healthcare, Chicago, Illinois, USA) yielding a sterile final product of highly purified leukocytes with a volume of approximately 45 mL.

#### Bedside leukapheresis of the donor prior to organ procurement—LEU

When the criteria for accepting a deceased donor, including research purposes, were fulfilled, an apheresis team, consisting of one surgeon and one apheresis nurse practitioner, was called to the donor hospital, bringing with them a mobile Spectra Optia (Terumo BCT Colorado, USA). A 14 Fr central dialysis catheter (CDC) (Med star, Columbia, Maryland, USA) was inserted using ultrasound guidance into the jugular or femoral vein. The leukapheresis procedure was thereafter performed according to the local PBMC collection protocol (CMNC protocol and following SOP IMT3127 Karolinska University Hospital).

Owing to time constraints set by following the organ procurement process, the total time of leukapheresis varied (122–259 min see Table 4). However, the aim was to obtain the maximum yield of target cells (PBMCs). After leukapheresis, the apheresis product was then transported to the Karolinska University Hospital at 2°C to 6°C and characterized cell numbers and composition at the Karolinska Stem Cell Laboratory.

### Cell Counting and Lymphocyte Characterization

All components were assessed for total WBC count using a Sysmex-XP300 (Sysmex Europe, Norderstedt, Germany) hematology analyzer and further analyzed for lymphocyte subsets using flow cytometry (DxFlex, Beckman Coulter Biotechnology, Suzhou, China). Cells were briefly re-suspended in PBS (Thermofisher, Waltham, Massachusetts, USA), supplemented with 0.4% Alburex (CSL Behring, King of Prussia, Pennsylvania, USA), and stained with fluorescence conjugated monoclonal antibodies for 10 min at room temperature (RT) and then hemolyzed using lysis buffer (IOTest3, Ref A07799, Beckman Coulter, Marseille, France) for 10 min. The cells were then kept at 4°C and analyzed within 1 hour. T- B- and natural killer (NK) cells were stained using a commercial antibody cocktail (tetra-CHROME, ref:6607073, Beckman Coulter, CA, USA), with CD45-FITC, CD3-PC5, CD19-ECD, CD56-RD1, as well as CD16-PE (Ref 332779 Becton Dickinson, San Jose, USA). T-cell subtypes were stained using a cocktail (tetra CHROME, ref:6607013) with CD45-FITC, CD3-PC5, CD8-ECD, and CD4-RD1. Cell viability was assessed using 7AAD (Ref A07704, Beckman Coulter, Marseille, France).

All CD45+ cells were identified as leukocytes. Lymphocytes were defined by high expression of CD45 and low granularity (low SSC). T-cells were defined as CD3+ lymphocytes and subtyped by expression of either CD4 or CD8. B-cells were defined as CD19+ lymphocytes and NK cells were defined as CD3-CD56+CD16+ lymphocytes.

### Statistics

All results are presented using descriptive methods. Data are expressed as mean ± standard deviation (SD) and range. Mean and SD were calculated using GraphPad Prism 9 (GraphPad Software Inc., San Diego, California, USA). Welch’s T-test was used to analyze the difference between 2 groups when there was only 1 variable. Analysis of correlation was performed using Spearman’s *r* test, and a *p* value of 0.05 or below was considered significant.

## Results

### Donor Characteristics

A total of 21 deceased donors were included. Donor characteristics are shown in Table 1. There were no significant differences in donor age, weight, height, peripheral WBC levels, CRP, nor hemoglobin levels. Peripheral blood was obtained from donors to compare the leukocyte composition of the donor’s pre-cell-procurement and showed no significant differences among the groups.

**Table 1.** Characteristics of Included Deceased Donors and the Composition of Leukocytes in Peripheral Blood Preprocurement.

Donor characteristics	BCL (*n* = 7)	BCS (*n* = 4)	LEU (*n* = 10)	Statistical difference
Age (mean)	18–84 (60.9)	26–70 (57.5)	42–77 (60.84)	*n.s.*
Male, %	43%	100%	70%	
Weight, kg (mean)	43–96 (69.4)	64–108 (79.5)	61–128 (80.6)	*n.s.*
Height, cm (mean)	150–191 (172.6)	175–185 (180)	160–192 (178.7)	*n.s.*
Leukocytes, ×10^9^/L (mean)	5.1–22.6 (12.9)	8.0–10.7 (9.55)	2.7–20.1 (12.12)	*n.s.*
Hemoglobin, g/L (mean)	92–154 (120.4)	98–147 (122.75)	81–124 (107.4)	*n.s.*
CRP, mg/L (mean)	26–282 (131.1)	23–222 (118)	60–342 (165.5)	*n.s.*

There was no difference between the age, weight, nor composition of leukocytes and hemoglobin levels between the donors included in the different PBMC procurement methods (ANOVA Dunne’s multiple comparison).

### Total Cell Yield and Viability

The cell yield, viability, and procurement parameters of the 3 methods are shown in Tables 2 to 4.

**Table 2.** Parameters of PBMC Procurement of BCL.

Donor	Retrieved blood volume	Time until apheresis (hours) (time from blood procurement until start of apheresis)	Processed volume at apheresis (ml)	Final volume (ml)	Total WBC yield (× 10^9^)	PBMC yield (×10^9^)	Viability
D1	2,500	19	8,560	107	1.32	1.02	99%
D2	3,000	20	20,385	100	4.68	1.81	91%
D3	7,800	3	16,136	104	8.64	8.26	98%
D4	4,000	6	9,214	74	3.67	3.36	99%
D5	2,000	4	4,701	38	1.12	0.84	99%
D6	6,000	14	14,427	157	4.80	3.05	97%
D7	3,500	19	8,859	51	2.04	1.3	99%

Characteristics of donors included in the BCL group. Start volume is the total volume of procured blood during cold perfusion in the organ donation procedure. Storage time is the time from blood procurements until apheresis, that includes transportation time and storage time (2°C–6°C) before leukapheresis. Final volume is the volume in the collection bag after apheresis.

**Table 3.** Parameters of PBMC Procurement of BCS.

Donor	Retrieved blood volume (mL)	Time until ALSS separation (hours) (time from blood procurement until start of apheresis)	Processed volume for sepax (mL)	Final volume (mL)	Total WBC yield (×10^9^)	PBMC yield (×10^9^)	Viability
D8	400	18	88	44	3.39	0.50	96%
D9	300	18	86	45	4.01	0.95	97%
D10	400	3	84	45	1.22	0.92	99%
D11	400	4	99	44	1.16	0.40	99%

Characteristics of donors included in the LEU group. Leukapheresis duration refers to the time from which the filling of the collection bag initiates until the end of collection. Final volume is the volume in the collection bag after apheresis.

**Table 4.** Parameters of PBMC Procurement of LEU.

Donor	Leukapheresis duration (mins)	Processed volume (mL)	Final volume (mL)	Total WBC yield (×10^9^)	PBMC yield (×10^9^)	Viability
D12	231	14,794	220	19.78	17.91	97%
D13	259	16,703	252	28.49	11.88	97%
D14	122	7,891	115	4.28	2.88	98%
D15	136	7,609	120	1.54	1.33	98%
D16	140	8,576	126	11.40	7.40	95%
D17	150	9,620	135	6.69	4.10	95%
D18	180	10,734	165	14.56	11.69	99%
D19	150	8,691	135	7.66	6.79	99%
D20	154	9,965	143	3.45	2.93	99%
D21	140	8,986	108	6.09	5.90	99%

Characteristics of donors included in the BCS group. Storage time until apheresis includes transportation and storage time (at room temperature) before buffy coat separation and later PBMC isolation using ALSS.

The viability of the procured cells was assessed using flowcytometry and showed no difference between the 3 methods.

**Figure 1.**
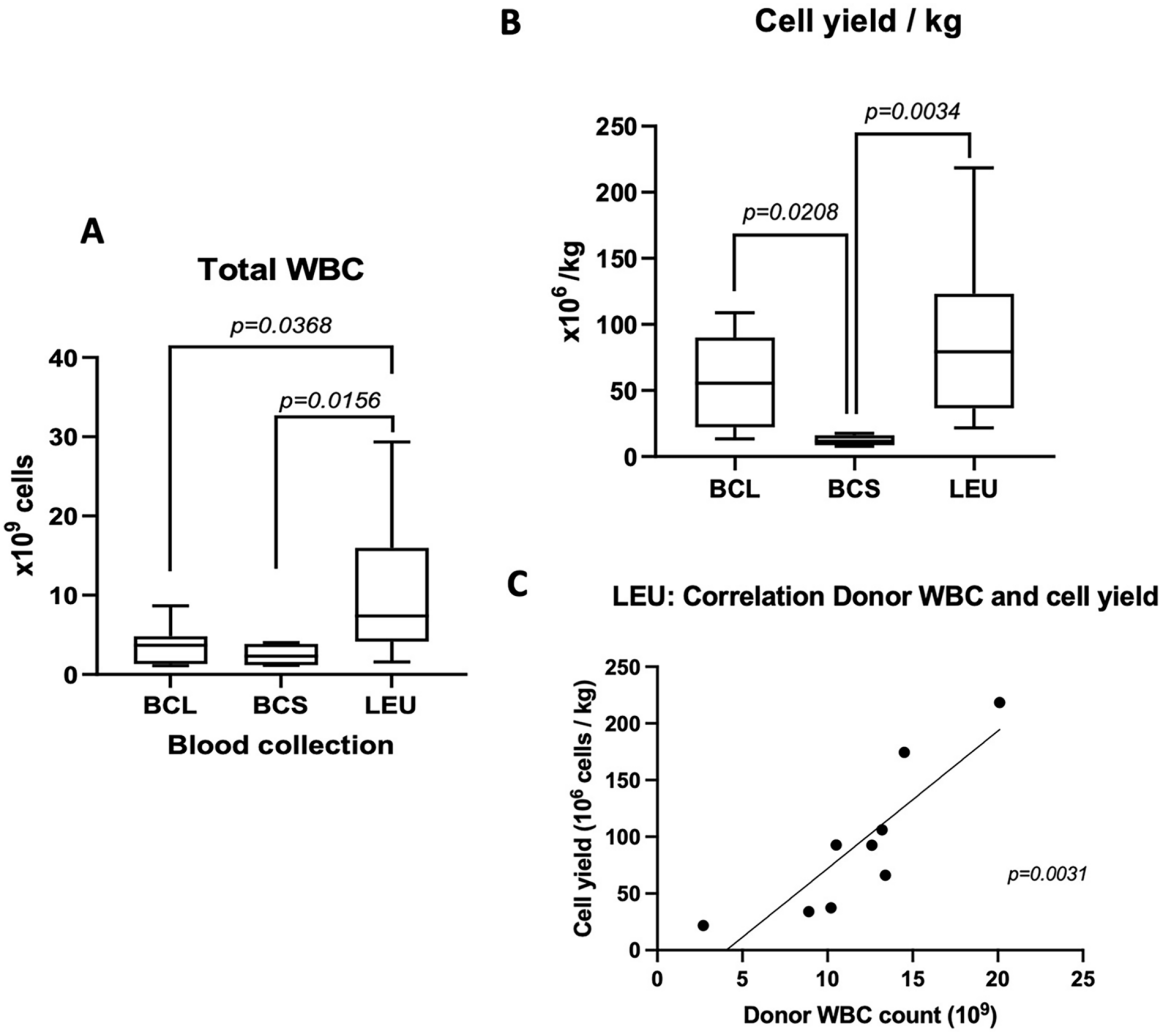
Cell yields and correlation to donor body weight. (A) Total yield of white blood cells between the 3 procurement methods and (B) cell yields 10^6^ cells/kg (body weight). Box floor and roof are the 25th and 75th percentile. Whiskers are the min and max values. Mean values are presented as the middle line in the boxes. Values of 2 groups are compared by Welch’s *t*-test. (C) The correlation between peripheral counts of white blood cells in ×10^9^/L (x-axis) and the cell yields (cells/kg) in the LEU group. There is a significant correlation between donor peripheral white blood cells and the cell yield (spearman *r* = 0.89, *P = .0031*).

The method with the highest cell yield was LEU 10.61 ± 8.61 × 10^9^cells (*n* = 10), compared to BCL 3.75 ± 2.63 × 10^9^ cells (*n* = 7, *P* = .0368), and BCS 2.44 ± 1.47 × 10^9^ cells (*n* = 4, *P* = .0156) (Fig. 1A).

When cell yield/kg (donor body weight) was compared, LEU had a higher average yield of 90.52 ± 63.09 × 10^6^ cells/kg, compared to BCS with 12.13 ± 4.05 × 10^6^ cells/kg, and BCL 57.64 ± 38.78 × 10^6^ cells/kg (Fig. 1B), though significant difference was found between LEU versus BCS (*P = .0034*) and BCL versus BCS (*P = .0208*).

There was a correlation between the WBC count in peripheral blood before the cell procurement in the donor and the total cell yield in LEU (Fig. 1D, Spearman *r* = .89, *P* = .0031). In contrast, the cell yield per body weight did not correlate with donor WBC (sample from peripheral blood) in BCL and BCS (data not shown).

### Leukocyte Composition

The percentage of cell fractions within the leukocyte compartment was compared. BCL and LEU had the higher percentage of lymphocytes, 58% ± 18%, and 46% ± 18%, respectively, compared to BCS with 21% ± 12% (BCL vs BCS: *P = .0157*). In contrast, monocyte percentage of LEU 29% ± 18% was significantly higher than BCL 15% ± 6% (*P = .0331*). The total acquired number of monocytes was also significantly higher in the LEU group (3.4 ± 3.0 ×10^9^) compared to BCL (0.52 ± 0.48 × 10^9^) and BCS (0.28 ± 0.15 × 10^9^) (data not shown).

BCS had a significantly higher percentage of granulocyte contamination, 60% ± 26%, compared to LEU 21% ± 17% (*P =,0456*) and a tendency of higher percentage of granulocytes compared to BCL 26% ± 22% (*n.s*.).

### Composition of Lymphocytes

Lymphocyte composition was further investigated by flow cytometry as shown in Fig. 3. BCL had a higher percentage of NK cells (49% ± 15%) compared to LEU (21% ± 10%) (BCL vs LEU, *P = .003*) and a tendency of a higher percentage of NK cells compared to BCS (24% ± 8%) (BCL vs BCS *n.s.*). LEU had a significantly higher percentage of B-cells (27% ± 11%) compared to BCL (12% ± 8%) *P = .0291* and a tendency of higher percentage of B-cells compared to BCS (18% ± 7%) *n.s.*

The average T-cell percentage was (57% ± 13%) in BCS, (50% ± 16%) in LEU, and (39% ± 15%) in BCL. CD4+ T-cell population amongst lymphocytes was (16% ± 8%) in the BCL group, which was significantly lower than BCS (34% ± 4%) (*P = .0038*), and LEU (34% ± 14%) (*P = .0080*).

### Safety Data and Adverse Events

None of the methods evaluated in this study led to an organ not being procured during the organ procurement process. Two livers were not procured due to cirrhosis and fibrosis and were deemed nontransplantable (Table 5) and were unrelated to the procurement of donor PBMC.

**Table 5.** The Intended Organs to Be Procured, Organs Not Procured and the Reason Why an Organ Was Not Procured.

Donor	Method	Intended organs to procure	Reasons for not procured organs	Additional comment
D1	BCL	Kidneys × 2, liver		
D2	BCL	Liver		
D3	BCL	Kidneys × 2, liver, pancreatic islets, heart valves		
D4	BCL	Kidneys × 2, liver, pancreatic islets		
D5	BCL	Kidneys × 2, liver, pancreas, heart valves		
D6	BCL	Kidneys × 2, liver, pancreas		
D7	BCL	Kidneys × 2, liver		
D8	BCS	Kidneys × 2, liver, heart valves		
D9	BCS	Kidneys × 2, liver, pancreatic islets		
D10	BCS	Kidneys × 2, liver		
D11	BCS	Kidneys × 2, liver		
D12	LEU	Kidneys × 2, liver		
D13	LEU	Kidneys × 2, liver, pancreatic islets, heart, lungs × 2		
D14	LEU	Kidneys × 2, liver, pancreatic islets		Atrial fibrillation of donor before organ procurement procedure. Assesed as unrelated to LEU procedure after evaluation by the apheresis department.
D15	LEU	Kidneys × 2, liver (for split), pancreatic islets, heart valves, lungs × 2		
D16	LEU	Kidneys × 2, liver	Liver assesed macroscopic cirrhotic	Not related to LEU procedure
D17	LEU	Kidneys × 2, liver, heart valves		
D18	LEU	Kidneys × 2, liver, pancreatic islets, heart		
D19	LEU	Kidneys × 2, liver, pancreatic islets, heart valves		
D20	LEU	Kidneys × 2, liver, heart valves, lungs × 2	Liver assesed macroscopic severe fibrotic	Not related to LEU procedure
D21	LEU	Kidneys × 2, liver, pancreatic islets, heart, lungs × 2		

None of the studied methods to procure PBMC lead to an organ not being successfully procured. The organs not procured were due to macroscopic assessed cirrhotic or fibrotic liver.

One donor developed atrial fibrillation before the organ donation procedure (D14), with a frequency of 110, that did not respond to cardiac resynchronization attempts. An amiodarone infusion was started, and the blood pressure remained stable. The fibrillation was resolved before the organ retrieval procedure and the event did not affect the organ procurement procedure. This event was assessed to be unrelated to the PBMC procurement procedure. Further investigations are required to identify the problems. There was one failure within the BCL group due to an urgent situation during the procurement procedure and the inability to cannulate the vena cava. This case was excluded from this study.

## Discussion

In this study, we compared 3 methods of retrieving PBMCs from deceased donors to be used as donor-antigen stimulators in the production of donor-specific regulatory cell therapies for tolerance-induction protocols. Our results show that procuring PBMC from the donor using any of these 3 methods did not affect the number of organs intended to be procured. The cell viability was comparable among the 3 methods. Considering the yield of leukocytes, both LEU and BCL were feasible for obtaining PBMC from deceased donors. LEU offers a controlled method to procure PBMC and has the highest procured cell yield of donor PBMC with a low amount of granulocyte contamination. BSC, on the contrary, which had difficulty gaining sufficient volume of blood, gave a lower cell yield and tended to a higher percentage of granulocyte contamination compared to LEU. Although the mean lymphocyte percentage of BCL was high (58%) compared to LEU (46%) and BCS (21%), approximately 50% of these were NK cells. The mean percentage of monocytes and B-cells, which are suitable for class I and II antigen presentation was higher in LEU compared to BCL and BCS.

For the majority of donor-specific regulatory cell products, large quantities of irradiated PBMCs are required for allo-antigen anergization^17^. Therefore, the use of relatively small quantities of peripheral blood drawn through the usual means may be inadequate. Other sources of PBMCs, for example, splenocytes, have been used in some culture protocols^5,18^. However, these methods may be challenged by the strict sterility requirements and preferred closed systems in Good Manufacturing Practices (GMPs) facilities. Comparing the methods we used, in this study, LEU gave the highest average yield of both total WBC and PBMCs. Methodologically, LEU is the most easily controlled cell procurement method, as the cell yield and composition can be evaluated during the leukapheresis procedure, for example, using an in-hospital cell counting system (SYSMEX) on an interim sample from the procured cells. If a too-low cell yield is discovered, more cycles of apheresis could be attempted in the PBMC procurement process. In contrast, in BCL and BCS, blood is collected during the organ procurement procedure and separated after transport back to the transplantation center for processing. If a low yield is achieved, there are no further options to increase the cell amount from a deceased donor. Another benefit of LEU is that the cell yield better correlates with the donor peripheral WBC count (Fig. 1C), which may help in the estimation of the cell yield before the cell procurement process.

Sterility is one of the corner stones of GMP production^19^. Therefore, sterile closed systems are preferred in the procurement of donor stimulator material. LEU collects blood via a CDC to a collection bag used, for example, in stem cell transplantation, which can be seen as a more strictly closed system. Using BCL or BCS, the surgeons cannulate the inferior vena cava, which considering the open abdominal cavity, may be a risky procedure in terms of pathogen contamination due to the open field, even if no viscera is perforated due to the open atmospheric field during open abdominal surgery.

Although the aim is to procure PBMCs, and cell separation methods strive for the highest purity of cell types, low-grade impurities may be difficult to avoid. In our case, common impurities are spillover cell lines, for example, erythrocytes, granulocytes, or platelets. Impurities may hamper the manufacturing process of an optimal product and are often included as required with limitations in release criteria specifications of cell products. In such cases, granulocytes are one of the presumed impurities, due to their fragile nature^20^, along with the risk of releasing pro-inflammatory mediators from their granule when disrupted^21^. The effect of granulocytes on adaptive immune responses has been discussed and shown mixed results^22,23^. However, neutrophil contamination could affect DC maturation in vitro and thereby their ability to activate T-cell proliferation^24,25^. This may obstruct an optimal manufacturing process, for instance with reduced viability of cultured cells or a reduced expansion rate of Tregs. Our results show that BCS yields a higher proportion of granulocytes compared to LEU and BCL. In BCS, the collected blood was centrifuged to reduce total blood volume and to concentrate the WBC component. Total blood volume in BCS was lower (400–500 mL) than BCL (2,000–7,800 mL); therefore, the centrifuge speed for concentrate WBC was selected aiming for collecting as much as leukocytes as possible. The final step of the cell purification process in BCS was using the ALSS. The ALSS preset cell purification protocol, which we could not alter due to company regulations, the whole WBC layer in the bag was collected. Therefore, the granulocyte percentage was higher, and consequently, the lymphocyte and monocyte percentage were lower in the BCS method compared to LEU. Regarding the lymphocyte composition, there were no significant differences between BCS and LEU. However, there was a numerically higher percentage of B-cells in LEU and a tendency of higher CD8+ T-cells in BCS compared to LEU. These differences were correlated to the individual donor’s peripheral blood composition of lymphocytes (data not shown), rather than the method of cell procurement.

Owing to the issues of the high granulocyte percentage, and the relatively low cell yield from BCS, we terminated the evaluation of BCS after 4 donors. However, our results show that BCS could be a feasible method to be used for procuring deceased donor PBMC in cell production protocols requiring lower amounts of donor PBMC. It is a simpler method compared to LEU. It requires very little hands-on training and is economically more beneficial. This is especially true if the PBMC separation protocol of the procured blood can be optimized to yield a lower degree of granulocyte contamination.

Another cell type that may affect the culture at high concentrations is the NK cell. NK cells have a capacity to secrete various cytokines under activation such as in the presence of non-self-major histocompatibility complex (MHC) molecules^26^ and thereby increase inflammatory responses. When the cell composition of blood was taken from a peripheral line from the donors, there were no significant differences among donors in these 3 groups (data not shown). The high amount of NK cells in the BCL group is in our belief caused by the pressure of perfusion solution through the capillary system and due to this, flushing out tissue resident NK-cells from the abdominal organs such as the liver. The high proportion of NK cells in liver perfusate compared to peripheral blood has previously been reported, and a higher proportion of NK cells in perfusate was shown to be associated with frequent incidents of acute rejections^27^.

**Figure 2.**
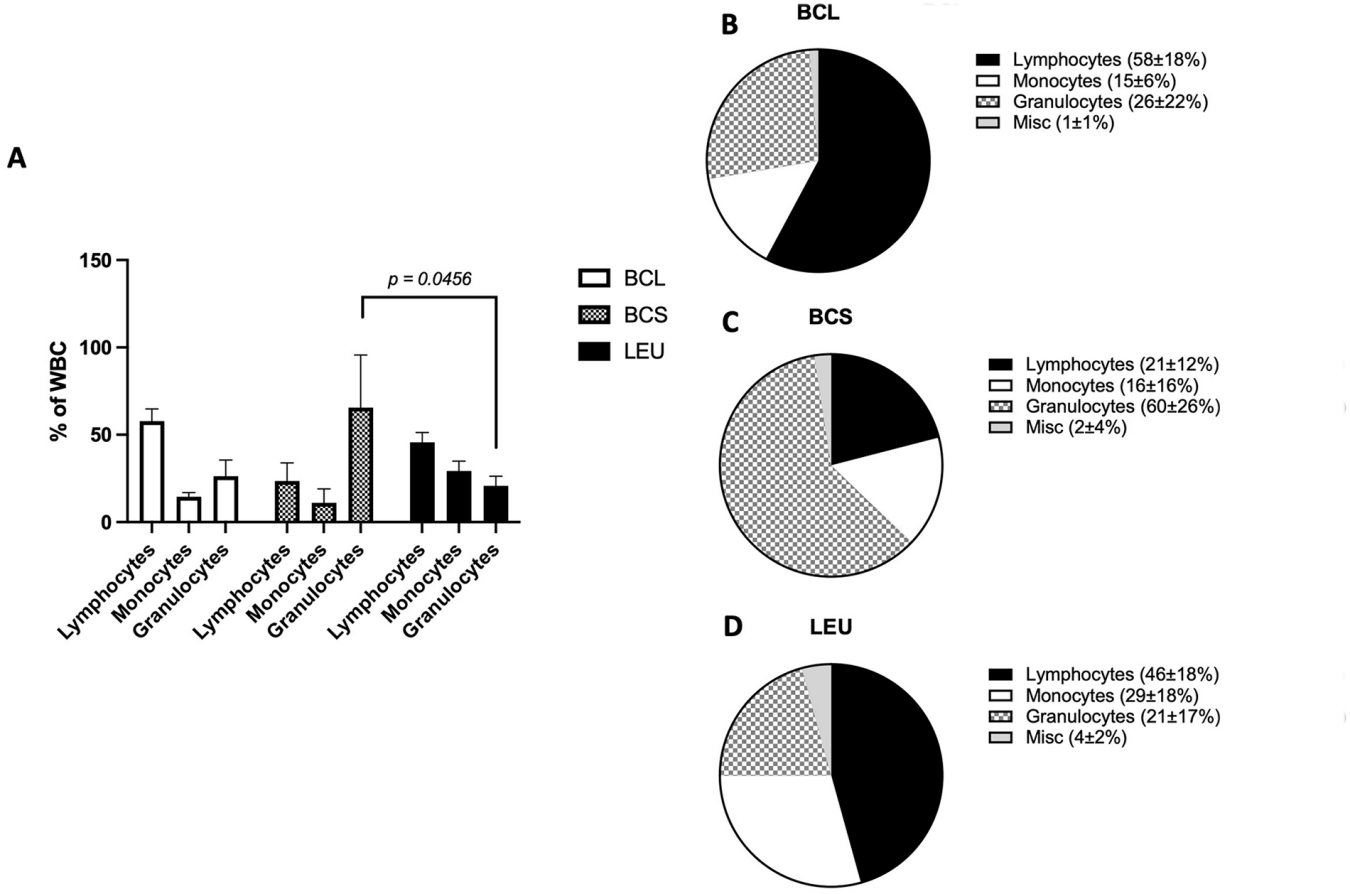
Composition of leukocytes. (A) The percentage of lymphocytes, granulocytes, and monocytes (in separate boxes within the box and whiskers) within the white blood cells. Values of 2 groups are compared by Welch’s T-test. (B–D) Pie charts of the distribution of white blood cells in the 3 different procurement methods.

**Figure 3.**
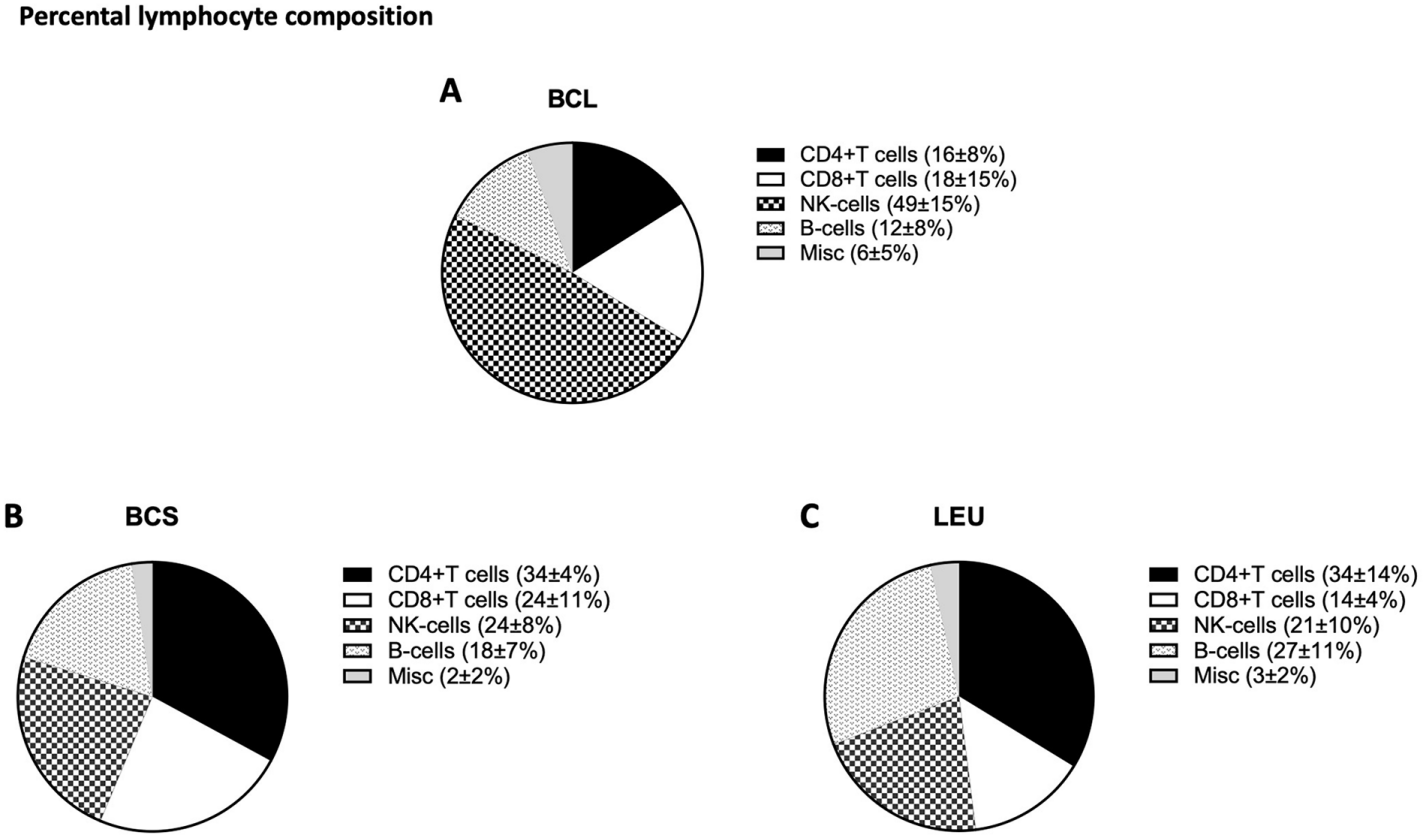
Composition of lymphocytes. (A–C) Pie charts of the distribution of lymphocytes in the 3 different procurement methods.

Although donor cells are irradiated when used as antigens, the residual donor NK cells may negatively affect the viability of the following cell production. The yield of monocytes was significantly higher in LEU compared to BCL and BCS, which in this case could be a benefit since monocytes present both HLA class II and HLA class I antigens. Monocytes express class I and II HLA antigens as well as costimulation molecules, which may control recipient CD4+ (and CD8+) T-cells. Cells separated by LEU contained a relatively high percentage of monocytes compared to cells separated by BCL and BCS (Figs. 2 and 3), which may be a benefit as a source of donor antigen presentation. Concordantly, B-cells which were in a relatively high percentage in LEU compared to the other groups also express both HLA class antigens and are also presumed useful for this purpose.

In our tolerance-induction protocol, recipient, and donor PBMC will be co-cultured as soon as these cells have been procured and transported to the GMP laboratory. The recipient will undergo the transplant operation on the same day. The cell production takes 14 days^5,28^, and the recipient will be treated with the cell product after GMP release at postoperative day 14. If BCL or BCS is used, there will be an extra step of PBMC separation after the procured donor blood is transported to the transplant center. This separation process takes additional time (in BCL the blood will be processed by a leukapheresis machine (Spectra Optia- Terumo BCT), while the blood procured by BCS will undergo buffy coat separation and PBMC purification using ALSS). Using the LEU method on the other hand will directly yield donor PBMC by leukapheresis in the donation hospital. These PBMCs can be directly used for cell production after irradiation. Hence LEU offers a quicker pathway from procurement to finalized raw material for cell production, which can also be seen as an advantage.

Regarding safety, none of the evaluated methods negatively affected the organ procurement procedure. There were 2 cases where the liver was not procured due to fibrosis and thereby deemed nontransplantable by the procuring surgeon. This was unrelated to the PBMC procurement procedures.

There was one case of atrial fibrillation of a donor after the LEU procedure. This event was assessed by the staff at the apheresis department and considered unrelated to the leukapheresis procedure. The donor was not an intended heart donor, and all the intended organs were successfully procured.

A major hurdle in manufacturing donor-specific tolerance-inducing regulatory cells is logistics. Several hospital units, for example, surgery, anesthesia, immunology, transfusion medicine, and GMP lab, are usually involved. In the living-donor transplantation setting, this can be scheduled ahead of time.

However, due to the unfixed occurrence of deceased donor transplants, a 24/7 on-call system needs to be in place. Using the BCL and BCS methods, it is sufficient for the organ procurement surgeon to be familiar with these blood procurement systems. The PBMCs can then be extracted from the procured blood in the transplant center at regular hours. In contrast, an apheresis team needs to be sent out to perform bedside leukapheresis in LEU, which is resource intensive. The development of centralized organ recovery centers may be an advantage when using this method.

One final important notice is the ethical legislation. This study was performed in Sweden, The Swedish Ethical Review Authority approved PBMC procurement using our described methods if the donors had expressed her/his consent (or the donors' relatives had interpreted the persons will to donate as positive) regarding the donation of organs and tissues for other medical purposes. Other countries may have different rules for organ donation and the use of organs or tissues for research purposes, which could complicate the implementation of these methods. However, this is not within the scope of this study.

In summary, this study evaluated the procurement of PBMC from deceased donors and showed that BCL and LEU are feasible methods. However, LEU enables the obtainment of large quantities of PBMCs in a controlled manner, with low-grade impurities in a sterile closed system. Leukapheresis is a well-documented and safe method to obtain WBCs^29–31^, and our evaluation did not show any major consequences of performing LEU bedside before the organ procurement procedure. Owing to this, LEU is the method we have chosen to proceed with for the development of our tolerance-induction protocol. However, the high resource demand, including the requirement of a trained apheresis team and a stable transportation system is a disadvantage. Additional studies evaluating the quality of the cell product that is generated using PBMC procured by these methods, and cost efficacy, will be necessary to determine the relative advantages of these different approaches.

Obtaining donor-specific PBMCs for regulatory cell treatment manufacturing is logistically troublesome. However, based on the encouraging results of regulatory cell tolerance-induction protocols in living-donor transplantation, it is important to pursue these strategies also for recipients of deceased donor transplantation.
